# How can knowledge exchange portals assist in knowledge management for evidence-informed decision making in public health?

**DOI:** 10.1186/1471-2458-14-443

**Published:** 2014-05-12

**Authors:** Emma Quinn, Carmen Huckel-Schneider, Danielle Campbell, Holly Seale, Andrew J Milat

**Affiliations:** 1 NSW Public Health Officer Training Program, Public Health Workforce and Training, NSW Ministry of Health, Level 7, 73 Miller St, North Sydney 2060, Australia; 2 The School of Public Health and Community Medicine, UNSW Medicine, Sydney, NSW 2052, Australia; 3 The Sax Institute, Level 2/10 Quay St Haymarket, Sydney, NSW 2000, Australia; 4 Centre for Epidemiology and Evidence, Population and Public Health Division, NSW Ministry of Health, 73 Miller St, North Sydney, NSW 2060, Australia

**Keywords:** Knowledge exchange, Evaluation, Collaboration, Online, Public health, Health policy, Information management

## Abstract

**Background:**

Knowledge exchange portals are emerging as web tools that can help facilitate knowledge management in public health. We conducted a review to better understand the nature of these portals and their contribution to knowledge management in public health, with the aim of informing future development of portals in this field.

**Methods:**

A systematic literature search was conducted of the peer-reviewed and grey literature to identify articles that described the design, development or evaluation of Knowledge Exchange Portals KEPs in the public health field. The content of the articles was analysed, interpreted and synthesised in light of the objectives of the review.

**Results:**

The systematic search yielded 2223 articles, of which fifteen were deemed eligible for review, including eight case studies, six evaluation studies and one commentary article. Knowledge exchange portals mainly included design features to support knowledge access and creation, but formative evaluation studies examining user needs suggested collaborative features supporting knowledge exchange would also be useful. Overall web usage statistics revealed increasing use of some of these portals over time; however difficulties remain in retaining users. There is some evidence to suggest that the use of a knowledge exchange portal in combination with tailored and targeted messaging can increase the use of evidence in policy and program decision making at the organisational level.

**Conclusions:**

Knowledge exchange portals can be a platform for providing integrated access to relevant content and resources in one location, for sharing and distributing information and for bringing people together for knowledge exchange. However more performance evaluation studies are needed to determine how they can best support evidence-informed decision making in public health.

## Background

With an increased awareness of the need for evidence-informed decision making in public health, knowledge management strategies need to be employed to ensure information is easily accessible [1], tailored and targeted [2], effectively disseminated [3] and shared among knowledge users [1].

Knowledge management is central to evidence-informed decision making, as it involves organisations and/or individuals creating, accessing, exchanging and translating knowledge (both explicit and tacit), usually in order to apply it to a particular policy or program challenge [4,5]. As the sheer volume of online information and knowledge resources increases, there is a need to have systems and tools that help policy, program and service decision makers manage this knowledge effectively [6-8].

Web portals are emerging as one information technology system capable of facilitating knowledge management, as they can help people to find the information they need, when they need it. Portals have evolved from simply being an efficient web tool for the one-way retrieval and transfer of information, to a platform for two-way collaboration and exchange among people in different organisations or professions, i.e. knowledge portals or knowledge exchange portals (KEPs) [9-11].

It appears that KEPs can facilitate knowledge management through three core activities, depending on their available design features and functions [11-13]: (i) knowledge access, by providing a single integrated point of access to a variety of relevant organisational or topic-specific information; (ii) knowledge creation, by creating and maintaining knowledge directories about portal generated content; and (iii) knowledge transfer and exchange, by facilitating information sharing and distribution and providing collaborative features that help to foster communities of practice.

A growing body of literature [14,15] suggests that communication technologies (e.g. video conferencing, virtual communities of practice and online interactive applications such as discussion forums and wikis) may be particularly relevant to the field of public health, where multidisciplinary team working and sharing of tacit and explicit knowledge is likely to increase the efficiency and effectiveness of policies, programs and services.

However, KEPs are a relatively new form of online communication technology and from a design perspective, it is still not clear which features and functions work best to support knowledge management in public health. We therefore conducted a systematic review of the literature to better understand the nature of these portals and their contribution to knowledge management in public health, with the aim of informing future development of portals in this field.

## Methods

### Scope

The review included analysis of the peer-review and grey literature to answer the following questions: 1) What are the common knowledge management design features of KEPs in the public health field? 2) How can KEPs assist in knowledge management for public health professionals? 3) Have KEPs been effective in terms of their uptake and facilitation of knowledge management in public health practice?

### Literature review

#### *Data sources and search strategy*

We conducted an aggregated search across multiple databases through Web of Science, ProQUEST and EBSCO, to identify potentially eligible peer-reviewed articles published between January 2001 and December 2013 and in English only. We searched all databases within the following meta-databases: (i) *ProQUEST Central*; (ii) *Web of Science Core Collection* and (iii) *EBSCO Host Research Collection* (a full list of databases searched is provided in Additional file 1: Table S1). The MEDLINE database was also searched. For the grey literature, we included grey literature databases available in the above meta-databases and conducted a search through Google Scholar. We also searched any publicly accessible portals identified from the peer-reviewed literature according to the definitions and inclusion criteria below (n = 7) (see Table 1).

**Table 1:** Publicly accessible portals as identified from the search of the published literature

**Portal name**	**Web address**	**Comments**	**Cited by**
European Union Public Health Information and Knowledge System (EUPHIX)	http://www.euphix.org/object_document/o4581n27010.html	Portal closed since 23 April 2012 – site still accessible	[28]
WhatisKTwiki	http://www.whatiskt.wikispaces.com/Publications		[24]
Canadian Best Practices Portal	http://cbpp-pcpe.phac-aspc.gc.ca/index-eng.html		[22]
National Collaborating Centre for Methods and Tools	http://www.nccmt.ca/registry/index-eng.html		[32]
Health Evidence Canada	http://health-evidence.ca/		[20,21]
Repository on Child Maternal Health	http://www.childhealthindiainfo.com/	No longer available through web address provided	[23]
Cancer Control Planet	http://ccplanet.cancer.gov/		[26]
Disaster Information Management Research Centre	http://disaster.nlm.nih.gov/		[29]

Both peer-reviewed databases and Google Scholar were searched with the following keywords and Boolean operators: (1) knowledge exchange portal OR web portal OR online OR website AND; (2) knowledge translation OR knowledge exchange OR knowledge transfer AND; (3) public health OR health.

#### *Selection criteria and classification of articles*

As there is no standard definition of a Knowledge Exchange Portal (KEP), we defined it broadly on the basis of two published definitions [16,17] as a web platform that enables a single point of access to information, applications and/or people (i.e. for knowledge exchange) in an organised manner for a specific target audience. For the purposes of this review, portals that functioned solely as interfaces for aggregated searching across multiple academic or library databases were excluded. However, portals that enabled aggregated database searching plus additional collaborative or interactive functionality tailored for a public health audience and for evidence-informed decision making (e.g. decision support tools, interactive tutorials etc.) were included. The KEPs of interest were those explicitly targeting public health practitioners and/or policy makers, either solely or as part of a broader target group. Public health was defined as aiming to prevent, promote or support the health of the population as a whole; this includes public health research, but not clinical medicine which is individually-based [18].

We included all articles (i.e. journal articles, studies, reports, conference presentations or abstracts, case studies and editorials) that described the concept, development, implementation or evaluation of a KEP in the field of public health. We set seven exclusion criteria in order to maintain a focus on web portals that could assist policy makers or practitioners in evidence-informed decision making in public health (as opposed to clinical or health business/administrative) decision making. Hence, articles were excluded if they described: (1) a business knowledge management tool designed to help internal employees navigate, understand and comply with organisational processes; (2) portals that aid in the management of health services or patient-specific data for the use of clinical decision-making; (3) online library catalogues; (4) portals delivering online education modules only (unless they include learning related to policy or practice decisions about public health); (5) the field of knowledge translation and/or knowledge exchange more generally and did not include information on KEPs; (6) portals designed to support consumer health decision-making for commercial products; and (7) portals designed to facilitate genomic or proteomic sequence searching and/or molecular visualisation. Issues over inclusion or exclusion of articles were resolved by discussion between three independent reviewers (EQ, CHS and DC).

#### *Type of analysis for review of articles*

As the articles were mostly descriptive, we analysed the content of the articles and synthesised the evidence according to the review questions above. Issues over discrepancies concerning analysis were resolved between three authors (EQ, CHS and DC) before reporting.

Where the articles described portal design features or functions, we classified these features into the domains of knowledge management as defined by Lee et al. [11] and Goh et al. [13]. This included: knowledge access features (mechanisms through which users access the portal and its information); knowledge creation features (processes whereby user information is captured and then stored and used for portal providers and users); and knowledge transfer and exchange features (mechanisms that allow the portal providers to foster user-to-user and provider-to-user sharing of knowledge) [11].

## Results

### Study selection and characteristics

**Figure 1:**
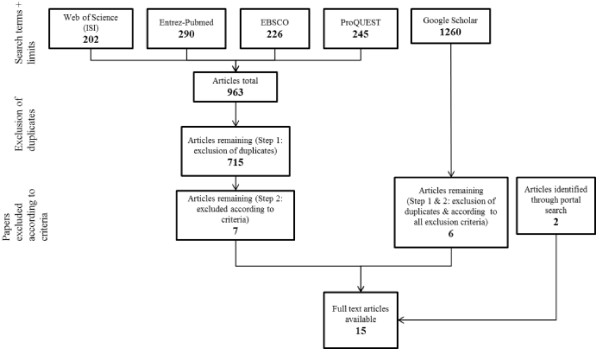
Summary of search strategy results for literature review.

The initial database and grey literature searches identified 2223 articles, of which 15 met the inclusion criteria (see Figure 1). Of the 15 identified articles, nine were published in peer-reviewed journals [19-27], four were conference abstracts [28-31] and two were online reports [32,33] identified from searching the publicly accessible portals (Table 1). Nearly all articles (n = 12) were targeting a public health audience [19-26,28,31-33], with the exception of three which specifically targeted a technology/information science audience [27,29,30]. Of the 15 articles, eight were case studies [21,22,26,27,29-31,33], six were evaluation studies [19,20,23-25,32] and one was a commentary/editorial article [28]. In these 15 articles, eight publicly accessible portals were discussed as listed in Table 1, two portals were each the subject of two articles i.e. Health Evidence [20,21] and the National Collaborating Centre for Methods and Tools (NCCMT) [25,32] and two portals cited in the literature were no longer accessible, either through discontinuation [28] or an incorrect web address [23].

### What are the common design features of KEPs in public health?

Only 11 articles [20,22-26,28-31,33] described portal features in sufficient detail to categorise them according to the knowledge management domains as defined by Lee and Goh (see below). Ten of these [20,22-26,28-31] mentioned features in the knowledge access domain, including search engines that enable free text or keyword searching of portal content, browsing features or site maps etc. Seven [20,23-25,28,30,33] mentioned a variety of features in the knowledge creation domain, mainly including sign up or registration features which enable portal providers to track members and their interests and also provide content tailored to their needs. Six [23,24,26,29,31,33] mentioned features in the knowledge transfer or exchange domain, mainly including collaborative features such as wikis, blogs and forums and online training video modules.

Khanna et al. [23] were the only authors to specifically use the criteria by Lee et al. [11] and Goh et al. [13] to conduct a ‘quality assessment’ of a portal in regards to features supporting knowledge management. Their assessment revealed that the maternal child health portal contained many features to support knowledge access, but not knowledge creation (e.g. sign up and user profiles) or knowledge transfer and exchange (e.g. blogs, instant messaging or online chat spaces etc.).

### How can KEPs assist in knowledge management in public health?

Twelve of the 15 reviewed articles [20-26,28,29,31-33] described the purpose and functionality of portals broadly in relation to the three domains of knowledge management [11,13] and are discussed here.

#### *Knowledge access*

KEPs can be a one-stop-shop for accessing systematic reviews of the evidence or synthesised evidence about the effectiveness of public health programs, interventions or policies [20-23,26] and are usually content specific e.g. related to chronic disease prevention [22], cancer prevention or control [26] and maternal and child health [23]. Maintaining the credibility, reliability and level of evidence accessible through these portals is particularly important. Several authors [21-23] in particular mentioned having robust content management strategies to ensure quick and easy access to the best-available evidence in one location.

KEPs can support knowledge access by providing an online registry of knowledge translation tools and methods. For example, the NCCMT portal [25] summarises knowledge translation tools that are designed to support evidence-informed decision making in public health. Furthermore, KEPs can also provide access to a range of epidemiological and demographic data to inform public health policy and program decisions at the national [28], regional or local level [29].

#### *Knowledge creation*

The creation of new knowledge generated from portal use is also a key aspect of how KEPs can facilitate knowledge management in public health. Other than by providing registration features which enable personalised search and content generation, users can create new knowledge by utilising other portal features. For example, the Online Health Program Planner [33] enables users to plan a health promotion program online based on best–practice frameworks and linked-in relevant evidence. The WhatisKT portal [24] enables users to contribute their knowledge on how to define key knowledge translation terms by posting information to the wiki in various content-specific spaces.

#### *Knowledge transfer and exchange*

KEPs can also be a platform for knowledge transfer via the automatic distribution of personalised (if user registration features exist) content e.g. email bulletins or newsletters etc. This is the case for portals containing online registries of public health evidence [20,21,26], or portals that send automatic and tailored content to users during a public health emergency [29] or disease outbreak [31].

KEPs can also be a platform for knowledge exchange by providing collaborative features (i.e. discussion forums) that enable communities or networks of practice to be established and maintained. ‘Communities of practice’ or ‘knowledge networks’ involve the sharing of explicit or tacit knowledge for a collective purpose. Three articles [24,27,32] describe how KEPs were designed with this purpose in mind, although their level of success was unclear.

### Have KEPs been effective in terms of uptake and/or facilitation of knowledge management?

In six articles classified as evaluation studies [19,20,23-25,32] the primary purpose was to conduct either a formative evaluation, i.e. report on user needs to inform the design of the portal [19,25,32] or a performance evaluation i.e. measuring usage once the portal [23,24] was accessible or measuring the association between portal use and evidence-informed decision making [20]. Two case study articles [21,22] also presented some evaluative information, one discussed a formative assessment of user needs in the design of the portal [22] and one article discussed usage data [21].

The three formative evaluation studies [19,25,32] involved consultation with user groups to understand what portal design features and types of functionality would aid policy makers and practitioners in their work. Atkinson et al. [19] found that users wanted easy access to sorted and rated published evidence, user-centred capability (e.g. tailored information and user support) and interactive features (e.g. connecting users with similar interests). Peirson et al. [25] evaluated the user needs of the NCCMT portal and found that users valued high volumes of content, useful links to external sites and summaries of evidence. They also found that users could be frustrated by search functions that were not user friendly or were time consuming. Forsyth et al. [32] found that the majority (>90%) of survey respondents who use the DialoguePH portal, anticipated new functionality through the portal not available elsewhere that would help them find tools and resources and training in the effective use of collaborative or interactive features. The one case study by Jetha et al. [22] reported that ~90% of users of the Canadian Best Practices Portal wanted easily accessible and summarised examples of best-practice in disease prevention and control and ~80% of users wanted links to other relevant websites.

Two evaluation articles [23,24] and one case study [21] reported on the performance of KEPs via web usage data on trends in the number, type, origin and retention of users over time. While Khanna et al. [23] and McKibbon [24] both demonstrated an increase in number of unique visits to their respective portals over time, the retention of users (i.e. high bounce rates and low proportion of returning visits) remained a problem. Dobbins et al. [21] reported a relatively stable number of total site visits for their target audience over time but with a substantial increase in time spent per visit (from 35 seconds to over 4 minutes), which they accredit to implementing more knowledge translation strategies e.g. tailored email updates, distribution of an electronic newsletter and making webcasts, webinars and videos accessible through the portal.

In terms of portals contributing to evidence-informed decision making (EIDM) in public health, Dobbins et al. [20] conducted a randomised controlled trial (RCT) with 108 regional public health units in Canada and investigated the use of three knowledge translation strategies on EIDM: (i) access to a KEP (HealthEvidence.ca); (ii) access to HealthEvidence.ca plus tailored electronic messaging (TM) and (iii) access to HealthEvidence.ca plus tailored messaging plus access to an organisational knowledge broker (KB). While none of these strategies had a significant impact on the primary outcome of EIDM, the second TM intervention group was associated with a significant increase in the use of evidence in recent public health policies and programs (p < 0.001) [20]. The impact of these interventions on ‘evidence use’ was modified by organisational culture [20], indicating that organisations with a low research culture favoured the KB intervention, whereas organisations with a high research culture benefited most from the TM intervention. In addition to this one research study, there were three further themes repeated throughout the literature related to the successful design and maintenance of KEPs to support knowledge management in public health as described below.

#### *Design and maintain the portal based on user needs*

Nine authors [19,21-23,25,26,28,30,31] emphasised the need to maintain a user-centric design philosophy over time. An assessment of user needs or requirements should be carried out initially to ensure the portal design is useful and usable for the target audience [19,25,26]. This initial step is also considered part of the process of gaining trust, acceptance and understanding of a new innovation or technology. Authors also reported the need to be flexible and adaptable in the design process [21,22] with ongoing user input [22,23] and to build on existing portal infrastructure [19,30]. In particular, having a robust content management strategy [28,30,31] that is responsive to user needs and ensures the reliability, credibility and accuracy of the information searchable through the portal is very important.

#### *Promotional strategies and funding partnerships for the portal*

Eight authors discussed the importance of promoting the portal to all user groups in order to generate and maintain use of the portal over time [21,23-27,32,33]. A well-defined and targeted promotion strategy can foster acceptance and instil confidence in users about the practical use of the portal. Promotional strategies can be direct to the target audience (e.g. emailing groups via listserves or providing flyers at conferences or events) or indirectly via partnerships with other key agencies [24]. Sourcing ongoing funding for the maintenance and technical development of the portal were also seen as important factors [21,27].

#### *Ensuring efficiency of knowledge transfer and exchange activity for users*

Three authors [23,27,32] specifically discussed the need to ensure that the economics of knowledge transfer and exchange (e.g. the benefit derived from community knowledge outweighs the time taken to write posts and participate in online discussions) were beneficial to users. Online communities of practice need to be unique and user-driven [32], have clear guidance and support to ensure group productivity [23] and require a balance between the number and skill of people involved [27].

## Discussion

While the published literature in this field is still evolving, this review demonstrates a wide variety of ways that KEPs can support knowledge management processes in public health. Public health KEPs may contain evidence libraries or registries, provide access to a range of epidemiological and demographic data, enable creation of new online content (e.g. health promotion plans) and/or maintain online communities of practice for sharing of knowledge about public health policies, programs or services. One RCT [20] has demonstrated that KEPs in combination with tailored messaging services can be effective at facilitating the use of evidence in policies and programs. However, there are several key factors that potential funders must keep in mind when designing and maintaining portals for a public health audience. Further robust performance evaluation of KEPs is needed to establish which design features best support knowledge management, as they can be a resource intensive investment.

In our review, we found that features supporting the process of knowledge access (e.g. key word searching of portal content) and knowledge creation (e.g. sign up and user profiles, as well as online planning tools) were the most popular design features in public health KEPs. The studies conducted by Lee et al. [11] and Goh et al. [13] previously confirmed this finding in a large sample of healthcare and government portals respectively across North America and the Asia Pacific. Lee et al. [11] further suggested that the design of KEPs could be improved by enhancing the number and type of collaborative features that support knowledge transfer and exchange. Formative evaluation studies identified in this review reported two common user needs for KEP design, easily accessible and searchable information [22,25] (preferably systematic reviews or quality assessed and summarised research studies or examples of best practice) and collaborative features (e.g. wikis, blogs, discussion forms etc.) that enabled targeted and interactive communication between public health policy makers and practitioners [19,32].

In terms of KEP usage or uptake, the reviewed studies revealed that while portal use may increase over time, retaining users remains a problem [23,24]. However by remaining responsive to user needs and providing active ‘push’ (e.g. tailored emails or newsletters) or ‘pull’ (e.g. content-specific webinars) knowledge translation or exchange functionality through the portal, funders or owners may entice repeat visits from users [21]. Furthermore, the utility of web usage statistics remains limited, unless longitudinal data are available to compare trends over time [34,35]. It has also been suggested [36] that web usage metrics for web portals should be different to that of websites and should include repeat use (same users accessing the portal), stickiness (length of time each user spends per visit) and frequency of use (number of unique visits per user per time period).

It is important to note that while web usage statistics provide a quantitative indication of knowledge access, monitoring usage patterns does not infer evidence-informed decision making in public health. Performance evaluation models that help to measure how portals are assisting in knowledge management activity [37,38] are still in development and no one model has been shown to be superior. Dobbin et al. [20] found that organisations with a high research culture responded best to the use of a KEP in combination with tailored and targeted messages, whereas organisations with a low research culture responded best to a knowledge broker in terms of facilitating the implementation of public health policies and programs. However it is important to remember that other barriers or facilitators for evidence-informed decision making exist [39,40] e.g. organisational culture, leadership, workforce and skills development etc.; and that KEPs are only one tool to link various silos of knowledge and people (internally within organisations and externally between organisations) and only one successful component of a knowledge management strategy.

This review also suggests some potential success factors that might be taken into consideration when designing, developing and maintaining a KEP for a public health audience. These include ensuring the portal has a user-centric design, promotional and funding strategies are sustainable and that online knowledge transfer and exchange activity remains beneficial for users.

### Limitations

Our search strategy was limited to articles published in English only. Given that the information communication technology sector is rapidly evolving in some developing countries (e.g. India, China and Brazil), we may have missed inclusion of articles published in other languages. Due to the difficulties associated with key word searching and comprehensively searching the grey literature, it is impossible to say we identified every eligible article for review; however our review process was systematic and covered a long time period. The findings of our review could be used with validity by public health staff that are considering designing and developing a KEP.

## Conclusion

KEPs can have design features that enable integrated access to relevant content and resources in one location, the sharing and distribution of tailored information and for bringing people together for knowledge exchange. Formative evaluation studies suggest that users want easily accessible and succinct information and collaborative features for knowledge exchange. Web usage studies reveal that while portal usage may increase over time, retention of portal users remains a problem. Evidence suggests that KEPs in combination with other knowledge management strategies can influence evidence-informed decision making in public health. However for the design of KEPs in this field to evolve based on the best available evidence, there needs to be more performance evaluation of KEPs in order to justify the resource investment over time.

## Abbreviations

KEP: Knowledge exchange portal; RCT: Randomised controlled trial; KB: Knowledge broker; TM: Tailored and targeted messages; EIDM: Evidence-informed decision making.

## Competing interests

Dr Holly Seale holds an NHMRC Australian based Public Health Training Fellowship (1012631). Payment for presentations: Dr Seale has received funding from Sanofi Pasteur, GSK and CSL Biotherapies for investigator driven research and for conference presentations.

## Authors’ contributions

EQ lead the design and conduct of the systematic literature review, including evaluating all articles for inclusion in the review, drafted the manuscript and finalised the manuscript with all authors for submission. CHS assisted in the conduct of the systematic literature review, including the evaluation of articles for inclusion in the review and in drafting and finalising the manuscript for submission. DC assisted in the conduct of the systematic literature review, including the evaluation of articles for inclusion in the review and in drafting and finalising the manuscript for submission. HS provided guidance in the conduct of the systematic literature review and participated in drafting and finalising the manuscript for submission. AM provided guidance in the conduct of the systematic literature review and participated in drafting and finalising the manuscript for submission. All authors read and approved the final manuscript.

## Pre-publication history

The pre-publication history for this paper can be accessed here:

http://www.biomedcentral.com/1471-2458/14/443/prepub

## Supplementary Material

Additional file 1: Table S1 List of individual databases searched through the online meta-databases*. Click here for file
