# Supplementary material for: How can knowledge exchange portals assist in knowledge management for evidence-informed decision making in public health?
Source: BMC Public Health. 2014 May 12;14:443. doi: 10.1186/1471-2458-14-443 (PMC4046147; doi:10.1186/1471-2458-14-443)
Supplement: Additional file 1: Table S1 — List of individual databases searched through the online meta-databases*. [file 1471-2458-14-443-S1.docx]

**Additional file 1: Table S1: List of individual databases searched through the online meta-databases***

| ****EBSCOHost Research collection:**** <http://www.ebscohost.com/> |
| --- |
| **Alternative Press Index Archive, America: History & Life, Anthropology Plus, Art & Architecture Complete, Art Index Retrospective, Audiobook, Avery Index to Architectural Periodicals, Book Review Digest Retrospective, Business Source Premier, CINAHL, Communication & Mass Media Complete, Communication Abstracts, Criminal Justice Abstracts, eBook Collection, Education Index Retrospective, Environment Complete, European Views of the Americas, Family & Society Studies Worldwide, Family Studies Abstracts, GalleryWatch CRS Reports, GreenFILE, Historical Abstracts with Full Text, History of Science, Technology & Medicine, Human Resources Abstracts, Index Islamicus, Index to Jewish Periodicals, Index to Legal Periodicals and Books (H.W. Wilson), Index to Legal Periodicals Retrospective, International Bibliography of Theatre & Dance with Full Text, International Political Science Abstracts, International Security & Counter Terrorism Reference Center, L'Année philologique, Left Index, Library, Information Science & Technology Abstracts, MLA Directory of Periodicals, MLA International Bibliography, Music Index, Peace Research Abstracts, Philosopher's Index, Public Administration Abstracts, Race Relations Abstracts, Readers' Guide Retrospective, Regional Business News, RILM Abstracts of Music Literature, Short Story Index Retrospective, SPORTDiscus, Urban Studies Abstracts, Violence & Abuse Abstracts, Women's Studies International** |
| **ProQuest Central:** [**http://www.proquest.com/**](http://www.proquest.com/) |
| [ABI/INFORM Complete](http://search.proquest.com.wwwproxy0.library.unsw.edu.au/abicomplete/advanced/fromDatabasesLayer?accountid=12763), [Accounting & Tax](http://search.proquest.com.wwwproxy0.library.unsw.edu.au/accounting/advanced/fromDatabasesLayer?accountid=12763), [Banking Information Source](http://search.proquest.com.wwwproxy0.library.unsw.edu.au/bankinginformation/advanced/fromDatabasesLayer?accountid=12763), [Canadian Newsstand Complete](http://search.proquest.com.wwwproxy0.library.unsw.edu.au/canadiannews/advanced/fromDatabasesLayer?accountid=12763), [CBCA Complete](http://search.proquest.com.wwwproxy0.library.unsw.edu.au/cbcacomplete/advanced/fromDatabasesLayer?accountid=12763) , [Hoover's Company Profiles](http://search.proquest.com.wwwproxy0.library.unsw.edu.au/hooverscompany/advanced/fromDatabasesLayer?accountid=12763), [OxResearch](http://search.proquest.com.wwwproxy0.library.unsw.edu.au/oxresearch/advanced/fromDatabasesLayer?accountid=12763), [Pharmaceutical News Index](http://search.proquest.com.wwwproxy0.library.unsw.edu.au/pharmanews/advanced/fromDatabasesLayer?accountid=12763) , [ProQuest Asian Business & Reference](http://search.proquest.com.wwwproxy0.library.unsw.edu.au/asianbusiness/advanced/fromDatabasesLayer?accountid=12763), [ProQuest Biology Journals](http://search.proquest.com.wwwproxy0.library.unsw.edu.au/biologyjournals/advanced/fromDatabasesLayer?accountid=12763), [ProQuest Career and Technical Education](http://search.proquest.com.wwwproxy0.library.unsw.edu.au/career/advanced/fromDatabasesLayer?accountid=12763), [ProQuest Computing](http://search.proquest.com.wwwproxy0.library.unsw.edu.au/computing/advanced/fromDatabasesLayer?accountid=12763), [ProQuest Criminal Justice](http://search.proquest.com.wwwproxy0.library.unsw.edu.au/criminaljusticeperiodicals/advanced/fromDatabasesLayer?accountid=12763), [ProQuest Education Journals](http://search.proquest.com.wwwproxy0.library.unsw.edu.au/education/advanced/fromDatabasesLayer?accountid=12763), [ProQuest European Business](http://search.proquest.com.wwwproxy0.library.unsw.edu.au/europeanbusiness/advanced/fromDatabasesLayer?accountid=12763), [ProQuest Family Health](http://search.proquest.com.wwwproxy0.library.unsw.edu.au/familyhealth/advanced/fromDatabasesLayer?accountid=12763), [ProQuest Health & Medical Complete](http://search.proquest.com.wwwproxy0.library.unsw.edu.au/healthcomplete/advanced/fromDatabasesLayer?accountid=12763), [ProQuest Health Management](http://search.proquest.com.wwwproxy0.library.unsw.edu.au/healthmanagement/advanced/fromDatabasesLayer?accountid=12763), [ProQuest Military Collection](http://search.proquest.com.wwwproxy0.library.unsw.edu.au/military/advanced/fromDatabasesLayer?accountid=12763), [ProQuest Newsstand](http://search.proquest.com.wwwproxy0.library.unsw.edu.au/newsstand/advanced/fromDatabasesLayer?accountid=12763), [ProQuest Nursing & Allied Health Source](http://search.proquest.com.wwwproxy0.library.unsw.edu.au/nursing/advanced/fromDatabasesLayer?accountid=12763), [ProQuest Political Science](http://search.proquest.com.wwwproxy0.library.unsw.edu.au/politicalscience/advanced/fromDatabasesLayer?accountid=12763), [ProQuest Psychology Journals](http://search.proquest.com.wwwproxy0.library.unsw.edu.au/psychology/advanced/fromDatabasesLayer?accountid=12763), [ProQuest Religion](http://search.proquest.com.wwwproxy0.library.unsw.edu.au/religion/advanced/fromDatabasesLayer?accountid=12763), [ProQuest Research Library](http://search.proquest.com.wwwproxy0.library.unsw.edu.au/pqrl/advanced/fromDatabasesLayer?accountid=12763), [ProQuest Science Journals](http://search.proquest.com.wwwproxy0.library.unsw.edu.au/sciencejournals/advanced/fromDatabasesLayer?accountid=12763), [ProQuest Social Science Journals](http://search.proquest.com.wwwproxy0.library.unsw.edu.au/socscijournals/advanced/fromDatabasesLayer?accountid=12763), [ProQuest Sociology](http://search.proquest.com.wwwproxy0.library.unsw.edu.au/sociology/advanced/fromDatabasesLayer?accountid=12763), [ProQuest Telecommunications](http://search.proquest.com.wwwproxy0.library.unsw.edu.au/telecomms/advanced/fromDatabasesLayer?accountid=12763), [Snapshots](http://search.proquest.com.wwwproxy0.library.unsw.edu.au/snapshots/advanced/fromDatabasesLayer?accountid=12763) |
| **Web of Science Core Collection:** [**http://wokinfo.com/nextgenwebofscience**](http://wokinfo.com/nextgenwebofscience) |
| Science Citation Index Expanded, Social Sciences Citation Index, Arts & Humanities Citation Index, Conference Proceedings Citation Index- Science, Conference Proceedings Citation Index- Social Science & Humanities, Book Citation Index– Science, Book Citation Index– Social Sciences & Humanities, Current Chemical Reactions, Index Chemicus |

* These databases were searched as they were available when using the standard settings in the meta-databases listed.
